# Comparison of response options and actual symptom frequency in the Japanese version of the Edinburgh Postnatal Depression Scale in women in early pregnancy and non-pregnant women

**DOI:** 10.1186/s12884-022-05257-y

**Published:** 2022-12-15

**Authors:** Hiromi Suenaga

**Affiliations:** Faculty of Health Sciences, Yamaguchi University Graduate School of Medicine, 1-1-1 Minamikogushi, Ube, Yamaguchi 755-8505 Japan

**Keywords:** Perinatal depression, Edinburgh postnatal depression scale, Pregnancy, Symptom frequency

## Abstract

**Background:**

The positive predictive rate of the Japanese version of the Edinburgh Postnatal Depression Scale (EPDS) is lower than those of other versions. This study aimed to confirm whether the EPDS Japanese version reflects actual symptom frequency and to examine the possibility of improving the positive predictive rate.

**Methods:**

This is a methodological study aimed at improving the positive predictive value of EPDS. The participants were 63 non-pregnant and 382 pregnant women. They answered the 10 questions of the Japanese version of the EPDS and reported the specific number of days as the frequency. The EPDS score (EPDS-S) and the frequency score (FREQ-S) were calculated for three factors of emotion: anhedonia, anxiety, and depression.

**Results:**

The positive rates of the EPDS-S and FREQ-S in pregnant women were 6% and 8%, respectively, which were lower than those in non-pregnant women (17%). On comparing the EPDS-S with the FREQ-S, a significant underestimation of frequency was observed in approximately 3% of pregnant women. The FREQ-S showed better internal consistency than the EPDS-S. Among the factors of emotion, women tended to rate anhedonia lower in the EPDS-S than in the frequency scale.

**Conclusion:**

Pregnant women tended to report a lower frequency on the Japanese version of the EPDS than their actual symptom frequency, which was especially true for those with a desire to self-harm. The combined use of the FREQ-S and EPDS-S can prevent underestimation and help improve the detection rate of depression.

## Background

Perinatal depression is the most prevalent complication related to pregnancy. Previous systematic reviews have reported the prevalence of perinatal depression to be between 13% and 19% worldwide [1–4]. The most recent report in Japan showed that the incidence of perinatal depression within 1 month postpartum and 1–3 months postpartum was 15.1% and 1.6%, respectively [5]. In addition, as many as 50% of women with postpartum depression experience depressive symptoms during pregnancy [6]. The recent coronavirus disease 2019 (COVID-19) pandemic has led to lifestyle and economic changes with an immense psychological impact, and an increase in the incidence of perinatal depression has been reported [7]. Moreover, changes in life, such as economic insecurity, partner conflict, and lack of exercise due to the COVID-19 epidemic, have been reported as a risk factor for perinatal depression [8]. Perinatal depression adversely affects children’s physical, psychological, and social growth and significantly affects a mother’s postpartum quality of life [9–13]. It has also been reported that perinatal depression attenuates dialogue and other contacts between mother and child [14]. Unchecked perinatal depression can lead to major depression and increase the risk of suicide [15]. The annual mortality rate due to suicide during pregnancy or within 1 year of delivery (the sum of deaths during pregnancy and late maternal deaths) is 8.7 per 100,000 deliveries (2005–2014) in the 23 wards of Tokyo [16], which is extremely high compared with other countries such as the UK (2.3 per 100,000 deliveries) [17] and Sweden (3.7 per 100,000 deliveries) [18]. In 2018, a study in Tokyo reported 63 cases of suicide during pregnancy and within the first year postpartum, over a 10-year period, and 60% of these cases were associated with mental illness, particularly perinatal depression [19]. It was reported that 5−14% of perinatal and postpartum women had thoughts of self-harm, and 20% of patients with suspected postpartum depression had thoughts of self-harm [20]. These findings have led the Japanese Society of Obstetrics and Gynecology to recommend the implementation of screening for depression in maternal health examinations since 2017. The Edinburgh Postnatal Depression Scale (EPDS), developed by Cox et al., is the most widely used screening instrument for postpartum depression [21]. It is a 10-item self-rating questionnaire, with each item assessing common depressive symptoms. The total score on the scale ranges from 0 to 30, with higher scores indicating a higher degree of depressive symptoms. There are a variety of cutoff points for the EPDS. According to the EPDS manual, 2nd edition [22], scores of 10 or more indicate high depressive symptoms, and a cutoff value of 10 is recommended for research use. The cutoff value for the Japanese version of the EPDS has been recommended by the Japanese Society of Obstetrics and Gynecology to be 9 points for postpartum pregnancy and 13 points for pregnancy, based on results of the studies by Usuda and Okano [2, 23, 24]. There is heterogeneity in the reported sensitivity and specificity of the cutoff scores among studies, which may be due to various factors, such as study methodology, language, or diagnostic criteria. The English version of the EPDS has a sensitivity of 86%, specificity of 78%, and positive predictive value of 73% [21], while the Japanese version has a sensitivity of 75%, specificity of 93%, and positive predictive value of 50% [23], indicating that the Japanese EPDS is less effective than the English version in identifying perinatal depression. Further, these results indicate that the positive predictive rate is lower when the Japanese version of the EPDS is used than in other countries that use the English version of the EPDS. Systematic reviews have shown that sensitivity, specificity, and positive predictive value remain uncertain across EPDS versions, even with differences in study design and cultural/language adaptation [25].

The low positive predictive value of the Japanese version of the EPDS may be because of the confusing and difficult-to-understand frequency of directly translated answer choices, possibly making it difficult to select the most appropriate option. In addition, the Japanese people have a reserved temperament due to their concern for social appearances. It remains unclear whether EPDS response options, translated into Japanese in a vague and nonnative tone, can accurately reflect the feelings of respondents. We frequently experience feedback from pregnant women after answering the EPDS that selecting the options in the answer sections is difficult. The vague wording may not reflect the personality of the respondent and may make accurate assessments difficult. Moreover, it has been reported that cultural differences can influence the choice of options in the context of the question, leading to response bias [26]. Currently, the low EPDS positive predictive rate among the Japanese may be because of the ambiguity of Japanese expressions, the reserved nature of the Japanese, or both. However, to the best of our knowledge, no reports have examined the causes of the low positive predictive rate. This study attempted to compare the frequency of the four traditional response options with the frequency of the number of days for which symptoms were reported by adding a column of the specific number of days to the responses for each item. Individuals might interpret the Japanese response options differently depending on their personalities, and a discrepancy might be found between the four Japanese options and the specific number of days. According to a recent cohort study, women are expected to have different anxiety factors due to changes in their living environment depending on their life stage, and maternal age has been reported to be associated with postpartum depression [27]. Therefore, it is also important to assess the association with age, which reflects life stage. In addition to the cultural differences in how Japanese women perceive the response options of the EDPS, there may also be differences in the emotional factors the scale measures. In fact, many systematic reviews of the EDPS have confirmed the multidimensionality of the scale. Several studies have identified two to four factor structures, including anhedonia, anxiety, depression, and suicide risk [28–33]. These results also suggest that the characteristics of the factor structure may differ depending on the perinatal period. If it becomes clear that there is a discrepancy between the Japanese version of the EPDS and the specific number of days, we can identify factors that cause the discrepancy between the frequency of the specific number of days and the conventional options, thereby gaining a detailed understanding of the factor structure specific to Japanese women, which will eventually contribute to improving the positive predictive value of the EPDS. It is hoped that this will eventually contribute to improving the positive predictive value of the EPDS. To the author’s knowledge, no study has compared responses to EPDS items with response frequencies on a specific day. The main objectives of this study were (1) to assess the agreement between response choices and exact symptom frequencies in the Japanese version of the EPDS, (2) determine if there is a discrepancy between the choices of pregnant women and the exact frequencies, and (3) improve the positive predictive value by identifying the causes of discrepancies when they occurred.

## Methods

This study is a methodological study aimed at improving the positive predictive value of EPDS.

### Participants

Participants were recruited between February 2020 and August 2021. The non-pregnant women included 63 female students from Yamaguchi University who were of reproductive age. The 20s age group was included in the comparison because this is the age at which women make many decisions about their future, and the diversity of their options causes anxiety. Furthermore, a total of 382 pregnant women between the ages of 18 and 43 years, in early pregnancy up to 15 weeks, were included in the study. Since this study included pregnant women in their teens to 40s, and the distribution of the number of participants in each age group was different, comparisons were made among the four age groups. All pregnant women were undergoing medical examinations at Tanaka Hospital, an obstetrics and gynecology clinic in Yamaguchi City. Tanaka Hospital, the facility where the study was conducted, is a specialized hospital in obstetrics and gynecology and has the highest number of births in the city, with approximately 500 births per year. The female students were asked to participate in the study via a mailing list, and their consent to participate in the study was obtained using a questionnaire. The pregnant participants were asked to sign a consent form after the study was explained to them in writing and verbal at the time of their antenatal checkup. All participants were informed that they could revoke their participation at any time after signing the consent form before data collection.

This study was initiated after receiving approval from the Ethical Review Committee of the Department of Health Sciences, Yamaguchi University in 2019 (control number: 600, 586-1), and it was conducted according to the Helsinki Declaration as revised in 1989. All collected data were kept anonymous and confidential.

### Exclusion criteria

Pregnant women with psychiatric disorders, including depression requiring medication, or with a history of diabetes, kidney disease, hypertension, heart disease, or multiple pregnancies that could pose a high risk for pregnancy and delivery were not included in the study because the study institution was a specialized obstetrics and gynecology hospital.

### Inclusion criterion

Pregnant women scheduled for antenatal care and delivery at the study facility were included. Participants included first-time mothers and pregnant women who had given birth.

### Procedure

Participants were asked to respond to the Japanese version of the EPDS, which was attached to a Google form set up in the cloud. The EPDS consists of 10 questions, each of which is rated on a scale of 0–3; the scores are summed to form the final EPDS score (EPDS-S), which ranges from 0 to 30.

For each of the 10 questions, a new item was added to record response frequency in days for the past week. Each statement in the EPDS has four response options (assessed on a Likert scale); items 3, 5, 6, 7, 8, 9, and 10 were scored in reverse. For each item, a frequency of 0 or 1 day was scored as 3 points, 2 or 3 days as 2 points, 4 or 5 days as 1 point, and 6 or 7 days as 0 points. Items 3, 5, 6, 7, 8, and 10 were scored in reverse, and the total score was used as the frequency score (FREQ-S). The cutoff value for both EPDS-S and FREQ-S was set at 13. Mean scores were calculated for each of the three emotional factors: anxiety (items 4 and 5), depression (items 7 and 9), and anhedonia (items 1 and 2).

Based on the results of the EPDS-S and FREQ-S of pregnant women, four groups were defined as follows: Group P was defined as positive results for both the EPDS-S, Group N as negative results for both the EPDS-S and FREQ-S, Group EP as positive results for the EPDS-S only, and Group FP as positive results for the FREQ-S only.

### Data analysis plan

Using descriptive statistics, variables were described as means and SDs or frequencies and proportions. The Mann–Whitney U test was used to compare the differences between pregnant and non-pregnant women and between the two groups. One-way analysis of variance was used for comparisons among the four groups, including the age of pregnant women, and multiple comparisons were also made using the Bonferroni correction. Spearman’s correlation analysis was used to examine the relationship between the EPDS-S and FREQ-S. Cronbach’s alpha and Spearman–Brown coefficients were used to analyze the reliability of both scores. All statistical analyses were conducted at a 5% level of significance, and a *p* value < 0.05 was considered significant. Statistical analyses were performed using JMP ver. 16.10 software (SAS Institute Inc., USA).

## Results

### Comparison between pregnant and non-pregnant women

Table 1 shows the distribution of the participants’ age and the number of pregnant and non-pregnant women in this study. All of the non-pregnant women (*n* = 63) were in their 20s, while the women in early pregnancy (*n* = 382) fell into four age groups: 10 − 19 years old (6), 20 − 29 (172), 30 − 39 (194), and 40 and above (10). The 10s were 18 and 19 years old, and none of them were under 16 years old. Both EPDS-S and FREQ-S were significantly lower in pregnant women than in non-pregnant women (Table 2). The overall EPDS-S positivity rate for pregnant women was 19%, which was significantly lower than that in non-pregnant women (Table 2). When comparing the EPDS-S and FREQ-S among each age group for pregnant women, the scores of the women aged 10 − 19 were significantly higher than those of other age groups, and there were no obvious differences among the other age groups (Table 2).

**Table 1:** Participant classification

	Non-pregnant women	Pregnant women	
*n*	63	Total	382
		10s (10 − 19 years old)	6 (1.6%)
		20s (20 − 29 years old)	172 (45.0%)
		30s (30 − 39 years old)	194 (50.8%)
		40s (40 − 49 years old)	10(2.6%)
Age, mean ± SD	20.2 ± 0.8		30.0 ± 4.7

*SD* standard deviation

**Table 2:** Comparison of the EPDS scores and frequency scores

EPDS-S (mean ± SD)	Non-pregnant women	Pregnant women					*p*-value
	*n* = 63	Total (*n* = 539)	10’s (*n* = 6)	20’s (*n* = 238)	30’s (*n* = 278)	40’s (*n* = 17)	
Positive rate	7.8 ± 4.6	5.1 ± 4.0***	9.3 ± 6.2	5.2 ± 4.0	4.9 ± 3.8	4.8 ± 5.4	< 0.0001
	0.17	0.06**	0.33	0.08	0.04	0.1	0.0069
FREQ-S (mean ± SD)							
Positive rate	7.4 ± 5.8	5.0 ± 4.8**	9.0 ± 7.5	5.1 ± 5.0	4.8 ± 4.4	5.6 ± 5.2	0.0028
						0.1	0.15

The *p*-values by one-way analysis of variance are shown. Multiple comparisons showed that EPDS-S and FREQ-S were significantly higher in the teens than in other age groups

**The *p*-value in comparison with the non-pregnant group was < 0.01

***The *p*-value in comparison with the non-pregnant group was < 0.001

*EDPS-S* Edinburgh Postnatal Depression Scale score, *FREQ-S* frequency score, *SD* standard deviation

**Fig. 1:**
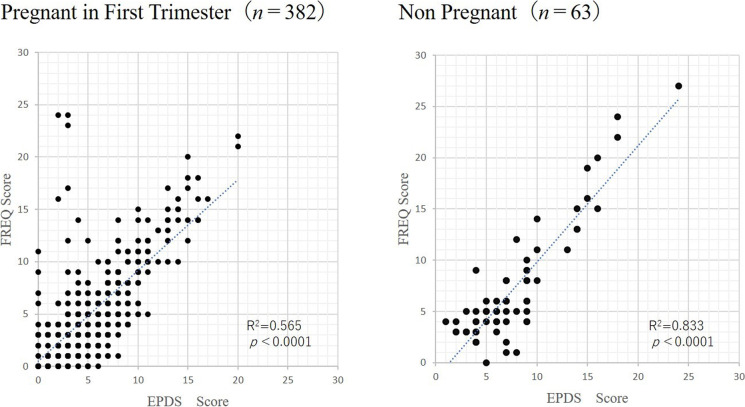
Associations between the total EPDS score and FREQ score. Left: pregnant women (*n* = 382); right: non-pregnant women (*n* = 63). EDPS: Edinburgh Postnatal Depression Scale, FREQ: frequency

The EPDS-S and FREQ-S showed a significantly positive correlation in both pregnant and non-pregnant women, although 3% of pregnant women underestimated the EPDS-S compared to FREQ-S, while a smaller percentage of non-pregnant women did (Fig. 1).

### Comparison of the three factors of emotion

While comparing the three factors of emotion, it was found that women tended to rate anhedonia lower than on the frequency scale (Table 3). The mean EPDS-S for each of the three factors of emotion (anhedonia, anxiety, and depression) was significantly lower for anxiety in pregnant women than in non-pregnant women and significantly higher for anxiety and depression in pregnant women aged 10 − 19 years than in other age groups. Among the three factors of emotion, the FREQ-S showed that the frequency of anhedonia was significantly higher among pregnant women aged 10 − 19 years than in the other age groups. Based on the results of the EPDS-S and FREQ-S of pregnant women, they were classified into four groups: group P (*n* = 53), group N (*n* = 288), group EP (*n* = 18), and group FP (*n* = 23). In the EPDS score, Group P had the highest values for all three factors of emotion among the four groups, although, in the FREQ-S, Group FP showed significantly higher anxiety (Table 4). 7% of pregnant women had a desire to self-harm, although this was not reflected in the EPDS-S of 68% of them. A desire to self-harm was present in 25% of the EP group, 50% of the FP group, 4% of the N group, and 40% of the P group. Anxiety and depression calculated by the EPDS-S were significantly higher in women with a desire to self-harm than in those without such a desire (anxiety: 1.12 ± 0.93 vs. 0.54 ± 0.65, *p* < 0.01; depression: 1.27 ± 0.16 vs. 0.61 ± 0.03, *p* < 0.0001). Furthermore, the FREQ-S also showed that the frequency of anxiety and depression was significantly higher in those with a desire to self-harm than in those without (anxiety: 1.92 ± 0.19 vs. 0.39 ± 0.03, *p* < 0.0001, depression: 1.69 ± 0.96 vs. 0.50 ± 0.57, *p* < 0.0001).

**Table 3:** Comparison of three factors of emotions obtained from the EPDS-S and FREQ-S by age

Score		Non-pregnant women	Pregnant women					
		*N*=63	Total (*n*=382)	10's (*n*=6)	20's (*n*=172)	30's (*n*=194)	40's (*n*=10)	*p*- value
EPDS-S	Anhedonia	0.19±0.40	0.13±0.31	0.33±0.52	0.11±0.29	0.14±0.32	0.20±0.42	0.25
	Anxiety	1.14±0.54	0.70±0.64***	1.28±0.71	0.74±0.62	0.66±0.62	0.63±1.10	0.0001
	Depression	0.74±0.07	0.58±0.03*	1.17±0.96	0.58±0.55	0.57±0.50	0.53±0.07	0.0247
FREQ-S	Anhedonia	0.73±0.08	0.37±0.03***	0.83±0.98	0.33±0.56	0.40±0.60	0.25±0.52	0.0001
	Anxiety	0.81±0.82	0.62±0.73	1.00±0.82	0.63±0.75	0.59±0.67	0.87±1.18	0.15
	Depression	0.52±0.70	0.48±0.61	1.11±1.09	0.51±0.64	0.45±0.56	0.53±0.48	0.12

The *p*-values by one-way analysis of variance are shown. The results of multiple comparisons showed that EPDS-S and FREQ-S were significantly higher in the teens than in other age groups

**The *p*-value in comparison with the non-pregnant group was < 0.01

***The *p*-value in comparison with the non-pregnant group was < 0.001

*EDPS-S* Edinburgh Postnatal Depression Scale score, *FREQ-S* frequency score

**Table 4:** Comparison between the four groups of pregnant women based on the EPDS-S and FREQ-S

		N (*n* *=* 346)	EP (*n* = 4)	FP (*n* = 12)	P (*n* = 20)	
EPDS-S	Anhedonia	0.09 ± 0.26	0.87 ± 0.63	0.13 ± 0.31	0.63 ± 0.46	*p*<0.0001
	Anxiety	0.47 ± 0.58	1.00 ± 0.71	0.79 ± 0.58	1.90 ± 0.60	*p*<0.0001
	Depression	0.55 ± 0.50	1.63 ± 0.48	1.04 ± 0.54	1.63 ± 0.53	*p*<0.0001
FREQ-S	Anhedonia	0.31 ± 0.55	1.13 ± 0.85	0.67 ± 0.62	1.13 ± 0.60	*p*<0.0001
	Anxiety	0.31 ± 0.55	0.75 ± 0.65	1.96 ± 0.96	1.85 ± 0.76	*p*<0.0001
	Depression	0.42 ± 0.47	1.38 ± 0.48	1.79 ± 0.69	1.83 ± 0.49	*p*<0.0001

The *p*-values represent the results of one-way analysis of variance among the four groups. *N* negative for both scores, *EP* positive for EPDS-S only, *FP* positive for FREQ-S only, *P* positive for both scores (cutoff value = 13 points)

*EDPS-S* Edinburgh Postnatal Depression Scale score, *FREQ-S* frequency score

### Reliability of the EPDS-S and FREQ-S

Table 5 shows the internal consistency of the EPDS items, as well as the EPDS-S and FREQ-S. The FREQ-S showed slightly better reliability than the EPDS-S (Cronbach’s alpha: 0.81 vs. 0.78). The Spearman–Brown coefficients were 0.87 for the FREQ-S and 0.84 for the EPDS-S, indicating good split reliability of the scales.

**Table 5:** Cronbach’s values for the EPDS items

	EPDS-S Cronbach’s αwithout the item	FREQ-S Cronbach’s αwithout the item
1. I have been able to laugh and to see the funny side of things	0.76	0.79
2. I have looked forward with enjoyment to things	0.76	0.79
3. I have blamed myself unnecessarily when things went wrong	0.73	0.79
4. I have been anxious and worried for no good reason	0.73	0.76
5. I have felt scared or panicky for no very good reason	0.74	0.77
6. Things have been getting on top of me	0.76	0.79
7. I have been so unhappy that I have had difficulty sleeping	0.76	0.78
8. I have felt sad or miserable	0.72	0.76
9. I have been so unhappy that I have been crying	0.76	0.78
10. The thought of harming myself has occurred to me	0.76	0.79
Cronbach’s α	0.78	0.81
Spearman–Brown coefficient	0.84	0.87

*EDPS-S* Edinburgh Postnatal Depression Scale score, *FREQ-S* frequency score

## Discussion

Pregnant women in this study received prenatal checkups at Tanaka Hospital, one of the obstetrics and gynecology facilities with the largest number of births in the prefecture, handling more than 500 births per year. Since the participants were patients of an institution with a large number of births but a small proportion of high-risk cases, the results were considered representative of the general population. The pregnant women who visited the facilities where the study was conducted were considered a surrogate population for the majority of low-risk pregnant women with no history or underlying medical conditions. Furthermore, as the EPDS scores for pregnant women were collected at the time of their first pregnancy checkup, they were able to respond to the EPDS without the influence of preconceptions and habituation in the same manner as non-pregnant women. The purpose of this study was to find errors arising due to the level of comprehension of the response options by determining whether there was a discrepancy between the response options selected by the participants and the specific number of days reported by the participants. The EPDS-S and FREQ-S both showed that non-pregnant women scored higher than pregnant women overall and pregnant women in their 20s, indicating that early pregnancy is a relatively less stressful situation than a regular life for female students in their 20s. However, it was clearly lower than the previously reported EPDS positivity rate in pregnant women [2, 23, 24]. The reason for the low positivity rate in this study might be that it was conducted in a general obstetrics and gynecology department. In contrast, most of the previous studies were conducted in populations with many high-risk cases, such as in university hospitals. The age-specific comparison of pregnant women showed that women aged 10 − 19 years had significantly higher EPDS-S and FREQ-S than the other age groups; this finding was consistent with previous reports that young pregnancy is a risk factor for perinatal depression [34]. This may be because teenage pregnancies are usually unexpected and are accompanied by social and economic instability. Both non-pregnant and pregnant women showed a good correlation between the EPDS-S and FREQ-S, although 10% of pregnant women had conflicting scores between the two. In particular, 3% of pregnant women showed an underestimated negative EPDS-S, and those with a markedly positive FREQ-S indicated a desire to self-harm. These corresponded to 21% of those who said that they had a desire to self-harm, while the other participants with a desire to self-harm showed either negative (46%) or positive (29%) results on both scores. 7% of pregnant women had a desire to self-harm, of which 68% had negative EPDS-S results, and 21% had positive FREQ-S results alone. These results suggest that it is important to note that the EPDS-S is often underestimated in pregnant women who have experienced a desire to self-harm since this desire is one of the symptoms of patients with depression. The conflicting results between the two scores may have been because of the difficulty in understanding the Japanese translation of the response options or selection errors on the part of the participants. However, caution should be practiced in interpreting these results since 5−14% of perinatal and postpartum women and 20% of patients with suspected postpartum depression reported having thoughts of self-harm [20]. The EPDS is evaluated using a cutoff value for the total score, but caution should be exercised in evaluating only the total EPDS score, as EPDS results are often low, even if the participant’s desire to self-harm is indicated.

Several studies have investigated the factor structure of emotions in the EPDS [28–33]. In this study, the emotional structure was broken down into three components: anhedonia, anxiety, and depression, with methods that have been reported to be comparable by the gestation period [35]. In a comparison of the three factors of emotion in early pregnancy, the EPDS-S showed similar values to those reported in previous studies [35], and anhedonia showed significantly lower values than the other two factors. In contrast, the FREQ-S did not differ among the three factors, suggesting a tendency to underestimate the items indicating anhedonia. This inclination may be responsible for Japan’s lower positive neutral rate and sensitivity compared with that in other countries.

The internal consistency in the current study was 0.78, which was comparable to previous results from overseas [21] but clearly lower than those in other Japanese reports [23, 36]. There may be a difference in sampling results between facilities with a large number of high-risk patients, and those results may be typical of the general population. The effect of Japanese characteristics and the ambiguity of the Japanese version of the choices cannot be denied in this regard, as the internal consistency of the EPDS-S was slightly lower than that of the FREQ-S.

### Limitations

All pregnant women in this study were in their first trimester of pregnancy; therefore, we did not obtain data throughout their pregnancy and the postpartum period. In addition, it is necessary to accumulate data for the second trimester of pregnancy, the second trimester, and the postpartum period. According to one study, several risk factors are associated with postpartum depression early in the first trimester of pregnancy [37]. Hence, further evaluations at different gestational periods and during the first year postpartum are needed. Longitudinal studies assessing the outcome of depression onset should also be conducted.

## Conclusion

The Japanese version of the EPDS tends to underestimate the actual frequency of pregnant women’s symptoms. Furthermore, participants who reported a desire for self-harm were often included in those with negative results, suggesting the need to focus on the idea of self-harm as well as the total score. In addition, because frequency expressions in Japanese may be affected by the respondent’s personality and symptoms due to their ambiguity, a more accurate diagnosis rate may be obtained by answers based on the specific number of days. Therefore, In the Japanese version of the EPDS, the combined use of the FREQ-S may help prevent underestimation and improve the positive predictive value of the scale.

## Data Availability

The datasets used and analyzed during the current study are available from the corresponding author upon reasonable request.

## Abbreviations

COVID-19: Coronavirus disease 2019
EPDS: Edinburgh Postnatal Depression Scale
SD: Standard deviation
EPDS-S: EPDS score
FREQ-S: Frequency score
